# A Community-Led Social and Emotional Well-being Trial Intervention With First Nations Male Parents in Remote Australian Indigenous Communities

**DOI:** 10.1177/15579883251365069

**Published:** 2026-02-18

**Authors:** Lyndon Reilly, Mick Adams, Alvin Kuowei Tay, Preston Deemal, Byron Diamond, Derrick Silove, Susan J Rees

**Affiliations:** 1 School of Clinical Medicine, University of New South Wales, Randwick, Australia

**Keywords:** First Nations, men, parenting, social and emotional well-being

## Abstract

Australian First Nations men are a specific and neglected demographic group at substantially higher than average risk for mental disorders, trauma, and suicide. A mixed methods investigation, including a randomised, controlled feasibility trial was undertaken into the effectiveness of a First Nations designed and led men’s parenting and social and emotional well-being (SEWB) intervention. The aim was to test if the intervention group improved on two validated measures for SEWB, compared with the control group. The SEWB intervention known as Enabling Dads (ED) was conducted in three remote Aboriginal communities in Far North Queensland, Australia, with 68 adult men. Outcome measures included the Indigenous Growth and Empowerment Measure and the Kessler 6 Distress Scale (K6). Before the intervention, no significant differences were found between the control and intervention groups for any variables, indicating that the groups were comparable at baseline. Post-intervention, the ED intervention group showed notably but not significantly higher scores on theoretically germane items including feeling more energetic, having a sense of belonging in the community, more confidence, feeling centred and focused, and experiencing less anger compared to the control group. Qualitative data with the intervention group provided confirmatory and new insights that strengthen the quantitatively indicated benefits that men gained from the ED program. The conclusion is that a group-based parenting intervention for First Nations dads may improve important indicators of their SEWB.

## Introduction

Australian First Nations men are a specific and neglected demographic group at substantially higher than average risk for mental disorders, trauma, and suicide. These mental health outcomes are referred to in this paper as social and emotional well-being (SEWB), a term used by many Aboriginal and Torres Strait Islander people to describe interrelated social, emotional, spiritual and cultural well-being, recognising connection to land, sea, culture, spirituality, family and community impacts on mental health. A First Nations person’s SEWB is influenced by current policies and past events (Gee et al., 2014). First Nations men have twice the suicide rate of non-Indigenous men in Australia, and it is substantially higher in rural and remote areas (Phillips, 2009; Brown, 2001; Eckert et al., 2004; Inder et al., 2011). Yet, despite the importance of Indigenous autonomy in determining better mental health outcomes, limited robust evidence exists on the effectiveness of Indigenous designed and implemented SEWB interventions in remote areas, particularly focusing on improving the SEWB of the male parent. The lack of culturally appropriate empirical evidence available to First Nations people can jeopardise efforts to fund programs that communities have found relevant and effective, leading to a situation where inadequate programs are offered on a short-term sporadic basis. Our study applied a mixed methods (qualitative and quantitative) design to establish the effectiveness of Enabling Dads (ED) as a SEW intervention, and to generate sufficient evidence for future communities to ensure the ED program, if sufficiently valued, could be continued and upscaled (Reilly et al., 2023; Takona, 2024).

In Aboriginal and Torres Strait Islander communities, men have always had an important role in preparing young people to enter and succeed in adulthood. In contemporary society, this role extends to the well-being of adolescent boys and girls. There is a compelling literature concerning the health and mental health of Australian First Nations men and fathers, however, apart from our research, few studies have systematically examined the nexus between men as parents and SEWB outcomes for them related to the parenting role (Bulman & Hayes, 2011; Carter et al., 2017; Clifford-Motopi et al., 2022). Because of impacts including colonisation and the forced removal of children from families (stolen generations), men’s parenting roles and the confidence to proudly take that role have been eroded. The Stolen Generations refers to a policy in Australian history (from the mid-1800s to the 1970s) that directed Aboriginal children to be forcibly removed from their families. The children were forced to grow up in homes or foster homes that were non-Indigenous. Traditional cultures and languages were banned, and children experienced extremely high levels of physical, sexual and emotional abuse, which compound the trauma of being without family relationships, and undermined the parenting roles of mothers as well as fathers. Our ED project and its early planning phase garnered a high level of First Nations community support because ED resists and challenges negative perceptions of Indigenous men, a position compellingly articulated by Uncle Mick Adams, the third author (Adams & Coltrane, 2005; Adams et al., 2017).

ED was designed by the first author with other Indigenous community members to address this problem directly and improve SEWB, by way of restoring men's dignity and respect in their family and community. ED aims to do this by restoring men’s confidence in parenting, and we posit that change in self-perception and engagement with their parenting role will strengthen the father’s SEWB. Many men have been alienated from their traditional parenting role and may struggle to fully value the important role they play in supporting their children, partners and communities. Programmes focusing on parenting, including with fathers, have often ignored First Nations men (MacDonald et al., 2024). Only some First Nations-focused SEWB programmes have emphasised the strengths and knowledge of men, and fewer seek to empower dads directly by way of identifying current and historical forms of oppression that have interfered with their capacity or confidence to parent (Prehn et al., 2022; Rossiter et al., 2017).

Context is critically important in designing culturally congruent mental health interventions (Ball, 2009, 2010; Dennison et al., 2014; Jannesari et al., 2021; Reilly, 2021; Reilly et al., 2023; Reilly & Rees, 2018; Rossiter et al., 2017). Remote communities in Australia share some similar, as well as unique factors that can add to as well as reduce mental health risks and challenges for Indigenous men. First Nations people living in remote areas are exposed to stress and adversity associated with remoteness from health services and supports as well as structural inequities, unemployment, poverty, and the historical influences of trauma including stolen generations and loss of family and country. For men, there may be additional gendered reasons that may prevent them from seeking assistance for health and mental health concerns, and that would be compounded by living at a distance from male-centred health care (Mahalik & Di Bianca, 2021; McKenzie et al., 2022).

Too often in remote regions of Australia we see communities miss out on SEWB interventions because of factors such as poor evidence to support projects to be upscaled, geographic distance from regional sites rendering remote area projects too expensive, and low levels of investment in training local people to ensure projects are culturally appropriate and can be sustained by a ready resource of local people (Bishop et al., 2017; Parker, 2010). There is growing awareness, but too few systematically documented examples, of the need for local First Nations people to be designing, leading, and evaluating their own mental health interventions, and within that context, tailored interventions for specific at-risk groups, including men (King et al., 2009). Research to influence policy and bridge the gap in mental health (SEWB) includes ensuring that informing interventions are based on empirical evidence that is designed and implemented by First Nations people, particularly men. Strong evidence of what works for First Nations men, by way of empirical interventions, can then be scaled up by First Nations people to improve population-level mental health. Most existing intervention research and practice, however, focuses on approaches that are designed for non-Indigenous populations and that seek to improve mental health through emphasis on generic psychological stressors and coping (Bryant et al., 2021; O’Keefe et al., 2021). Western interventions may refer to, but often fail to address, the complex influences and historical traumas on First Nations mental health, and they are not culturally nuanced to resonate with or leverage the strength and knowledge of First Nations people (Bryant et al., 2021; Laird et al., 2021). In addition, many successful programs designed and led by First Nations people, that have strong Indigenous community support, lack empirical evidence which contributes to the challenges with attracting ongoing funding. This means that the best programs that should have been scaled up to improve mental health outcomes and ‘close the gap’ have failed to be supported and sustained (Fogarty et al., 2018; King et al., 2009; Laird et al., 2021; Pham et al., 2021; Wright et al., 2021).

We therefore used a randomised controlled feasibility trial and qualitative interview to examine the effectiveness (or otherwise) of a men’s parenting intervention, known as ED. We also anticipated that because of our ED First Nations designed, led and implemented intervention, and the related, newly developed local skills and capacities, there would be evidence of empowered community health workers ready to initiate and run ED into the future, and to trial other new SEWB projects. These outcomes would be clear examples of First Nations self-determination and autonomy in driving better mental health outcomes.

Our hypothesis was that participating men randomised to the ED intervention would demonstrate greater improvements on items of SEWB which are indicators of mental health status, compared with men randomised to the control group, and the ED group would feel more empowered by the parenting role that contributes to the well-being of their adolescent child, compared with those in the control group. We further hypothesised that men who participated in the ED intervention, compared with men in the control group, would feel more empowered and therefore become more engaged and connected with the broader community (Parke et al., 2006). In the next phase of data analysis from the study, we will examine if the men’s participation in the program has a corresponding benefit for their child’s SEWB. We will report those findings in a future paper.

## Methods

### Design and Setting

The intervention was conducted in 3 remote First Nations communities in Far North Queensland, Australia (Appendix A). These communities tend to have poorer health outcomes compared with urban and larger regional areas because of unique characteristics and challenges. Although the advantages of living in these communities are many, some factors associated with worse health and mental health outcomes in remote communities compared with regional towns and cities include unemployment, crowded housing, distance from health and mental services and supports, fewer sources of fresh and healthy food, and poor or restricted access to education for children and adults (Drew et al., 2022; Foster & Hall, 2021).

### Participants and Recruitment

We recruited 38 First Nations men in three remote area communities in the North of Australia. All participants were recruited by word of mouth, and all who volunteered to participate were included (apart from exclusions, see below). We revised our recruitment aim in response to concerns that the population sites were small (<1500 people in each community), and that we did not want any men who were keen to be involved to miss out. The aim was therefore to recruit as many men as possible within a 2-month recruitment period at baseline. Given the RCT was a pilot or feasibility design with small numbers, and we were using a mixed methods approach with a qualitative component, we considered based on similar studies that we would aim for 30 men from across the three sites (O’Cathain et al., 2015). Exclusion criterion included overt severe mental illness or being affected by alcohol at the time of the study. Men could be any age if they had an adolescent child in their care. We anticipated small numbers in our sample, so we did not aim to assess differences related to age. People in remote communities are all similarly on low income, so we did not assess differences according to economic status. We ensured that information describing the study intervention and control condition (for local research personnel, participants, and the wider community) was articulated in a way that would avoid creating the impression that one group condition was superior to the other. This veiled approach reduced the risk of bias regarding the value or expected efficacy of either of the two group conditions (Reilly et al., 2023).

Eligible men were randomised to receive the ED intervention or a usual men’s group (1:1 ratio). Randomisation was achieved using a block design by an independent statistician using STATA software (StataCorp). Training, reliability checks, fidelity of data gathering and entry were ongoing and iterative. The importance of privacy and confidentiality was emphasised in the men’s intervention groups. The men were reassured that they were not being judged as parents and that all data from interviews are de-identified for analysis.

Community-based researchers (CBRs) are First Nations people recruited from each local community site, with community health, welfare or mental health experience. The CBRs measured outcomes at baseline and endline. CBRs were fully trained and supported by the lead authors on-site and in Cairns, the closest regional city to the communities. Staff received ongoing support via regular time spent in communities by chief investigators, phone, and online using Microsoft Teams. Our CBRs and the larger team had regular meetings with key stakeholders including community members and Elders, Council and community-controlled health services.

**Figure 1.**
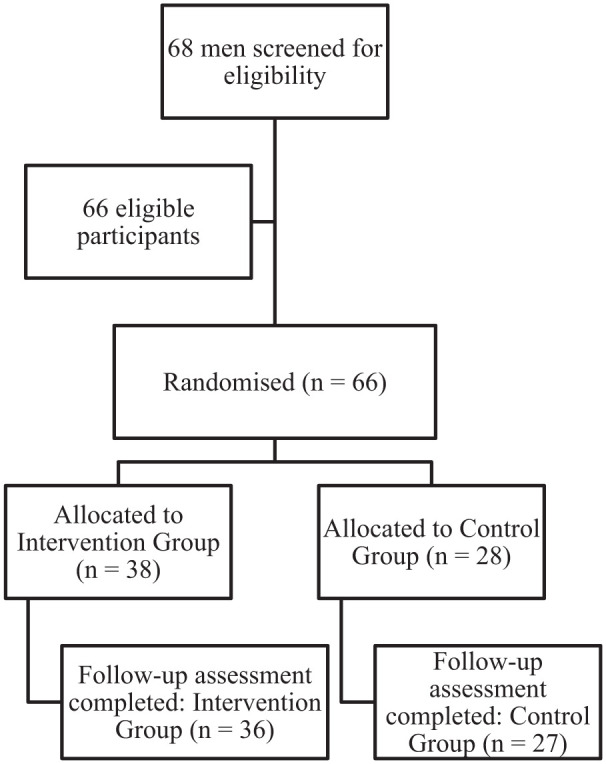
Participant Flow Diagram for the ED Trial. *Note.* ED = enabling dads.

Of the 68 men screened, 66 (97%) were eligible and enrolled in the study. Of those randomised, 95% completed the baseline assessment, and 95% completed the endline assessment (see Figure 1 for participant flow diagram). Qualitative data were collected in the months following the final interview with the ED participants, aiming to provide narrative insights to further explain the quantitative findings.

The intervention was a randomised controlled feasibility trial (RCT), using a novel and culturally designed and manualised psychoeducational parenting program with men, known as ED (Lukens & McFarlane, 2004). The comparison group received a culturally congruent and familiar yarning/relaxation (YR) condition. The ED intervention and the measures resonated with our informing theories, which were First Nations led, trauma-informed, and anti-colonial (Laird et al., 2021).

The outcome measures were selectively chosen because they contain key areas for evaluating changes in SEWB:

1. Growth and Empowerment Measure (GEM) (Haswell et al., 2010). The GEM measure is designed and validated with Australian First Nations communities. It was designed and applied in our study to assess the empowerment and mental health of First Nations men. On a five-point scale, questions include “I feel slack, like I can’t be bothered to do things even when I want to” at the bottom end of the scale, to “I am strong and full of energy to do what is needed” at the top of the scale.
2. The widely used Kessler 6 Distress Scale (K6) (Kessler et al., 2017), international measure for mental distress is validated with First Nations populations and is included in the GEM, including two additional items found to be relevant to Indigenous people: “happy in yourself” and “angry at yourself or others” (K6 plus 2).

CBRs facilitated the ED program in their community (three communities) for 1-hour sessions for 6 weeks. Each group involved no more than 10 to 15 men. The CBRs recruited the male participants into the program by word of mouth, and the use of snowball sampling where people share information with others about who might be interested (Etikan et al., 2016). The program was offered in a culturally familiar and valued men’s group format. We recruited 40 men across the three communities at baseline, after 50 men expressed an initial interest in the program. Due to COVID-19 and other illnesses, flooding events, Sorry Business (community ritual following a death), and employment needs away from the community, we recruited 40 men, and 38 men complete the whole program.

Baseline measures to assess the effectiveness of the intervention were completed following randomisation of the men. We did this to help ensure comparable groups at the outset and minimise the risk of selection bias. The person who undertook the baseline interviews was blinded to the condition or intervention. Follow-up interviews were undertaken between 1 and 2 weeks after the completion of the program.

A tested and community-validated manualised ED intervention was applied with the intervention group. The content topics included:

Session 1 – start-up session (welcome, validating who we are, ourselves, families, communities, histories, spiritual places, our country, why we are proud).
Session 2 – starting from dad’s strengths (what we do as men and why it is valuable for our kids, partners, families, communities). The men discuss and answer questions that reflect their learning about parenting, for example, “What are the barriers or problems that confront men as parents” and “what do men do when they are parents” and “Who should not be involved with parenting kids.”
Session 3 – understanding men’s parenting (what impacts from history, colonisation, Stolen Generations, and assumptions about men and their role as dads have influenced us being Dads and how we feel).
Session 4: homework – doing men’s parenting (what do Dads do, engaging with your children as their dad).
Session 5: sharing our homework (how did we go, reflecting on the homework tasks, and sharing experiences of being a dad).
Session 6: benefits of your parenting to you, your partner, and your community.

### The Comparison Group – Yarning Relaxation

The comparison Y/R condition was based on the tradition of yarning coupled with a component of relaxation training, commonly offered in the context of men’s groups. Men met for the same number and duration of sessions as the ED intervention in a safe, social setting where they were supported in yarning (discussing) issues that affect all aspects of their lives. The facilitator used general group support and group dynamic skills, encouraging mutual respect, listening, positive interactions, support, and the opportunity to express feelings and concerns without being judged or censored. The men could talk about any challenging issues, as was relevant to them at the time. Two structured and tested sessions of stress management are administered to the control group. In training the facilitator, with equal time and emphasis as training for the ED condition, there was no special focus on parenting, although spontaneous discussion of the topic was not discouraged. If a parenting-related matter arose, it was discussed generically without the empowerment focus on enablers and barriers.

### Cultural Appropriateness

We acknowledge that Western methods may not resonate or accurately reflect what is being measured. Our team works across cultures in the mental health field, and we take a systematic approach to ensure our methodology reduces the risk of misunderstanding and misinterpretation across cultures. Furthermore, every study is strengthened by future research to confirm findings or establish variance, and then to consider the variables that may account for differences, including that measures may not be determining variables as intended. Our commitment to this end is to document our process accurately, so that replication of the measurement and the intervention is possible, including a future randomised controlled trial, if considered appropriate.

Our study employed local people in each site to be trained as research assistants (CBRs), under the direct supervision of First Nations academics. The importance of locally trained people to develop and conduct the ED intervention and study cannot be understated. Advantages include appropriate cultural knowledge and advice from them to ensure the intervention resonates and is more likely to be effective; training and support for local people to support their careers; and capacity building amongst local people, which provides mental health skills and SEWB research knowledge to benefit communities into the future.

We used yarning circles, a culturally acceptable format, to gather data for this analysis.

Data sharing: An important feature of our study was that we take our findings back to Elders and councils in each community to share and discuss, a process which respects local Indigenous insights and considerations regarding the accuracy and usefulness of our findings before they are reported. Data collected in each community are also owned by that community, that is, we promote Indigenous data sovereignty.

### Positionality Statement

Our team and its capacity for this project is strengthened by Indigenous knowledge, lived community experience, and absolute commitment to genuine First Nations community engagement, ownership and leadership, including our employed research staff being all First Nations people from the participant communities. The first author designed this project. He is a proud First Nations Badtjala man and respected mental health researcher and practitioner, including in the regions of this study. The second author is connected directly to this region as a proud descendant of the Yadhiagana/Wathuti people from Cape York Peninsula. He is also a nationally and internationally acclaimed for his decades of work in Indigenous men’s health research and policy, Indigenous training, supervision, mentoring and community development. The last author is non-Indigenous, an internationally recognised leader in trauma and community mental health research, and she has lived and worked in Far North Queensland, including with First Nations communities, for more than 17 years.

### Analysis

We conducted a mixed methods study, involving the community-based and led feasibility RCT and a qualitative interview component of the ED intervention, undertaken with the ED group participants. We aimed first to determine the probable effectiveness of the intervention in improving men’s SEWB, as determined after combining the quantitative and qualitative findings and conducting a mixed methods analysis. The formative RCT study, we considered, would inform a future fully powered RCT, which would be compliant with CONSORT guidelines.

### Quantitative Analysis

The primary objective of the quantitative analysis was to assess whether participation in the ED intervention led to improvements in SEWB among First Nations male parents, compared to a control group. Analyses focused on two validated outcome measures: the GEM and the Kessler 6 Distress Scale (K6).

Descriptive statistics were used to compare mean pre- and post-intervention scores for both groups. Change scores (post minus pre) were calculated for each participant, and independent samples *t*-tests were conducted to compare these changes between the intervention and control groups. This approach was used to assess the overall effect of the intervention while reducing the risk of Type I error due to multiple comparisons.

All tests were conducted at a significance level of *p* < .05, and all reported values include 95% confidence intervals (CI).

### Qualitative Analysis

We conducted qualitative interviews with participants at the end of the 6-week program. All men were invited, and 15 participated in the qualitative interviews, conducted in Doomadgee and Hopevale communities. The man shared their perspectives on the program, held during men’s group meetings (Yarning Circles) and facilitated by our CBRs. The aim was to gather data on the men’s views on the intervention. Semi-structured questions included if the men felt the intervention was beneficial for their SEWB and parenting, and why, and what could be improved.

Data were recorded and transcribed, and NVivo software (QSR International) used for data management and analysis. Thematic analysis was undertaken by our First Nations authors and applied to deduce (discuss, consider, debate) the meaning of what was being shared. Two raters discussed each point and conferred regarding the intent and significance of it in the context of the quantitative data. The themes from this analysis are described below, with salient qualitative quotes from participant men.

Mixed methods analysis to examine where and how it confirmed or did not support the quantitative data was undertaken to provide integrated data to answer the research questions. The analysis involved a convergent parallel design, where qualitative and quantitative findings were discussed and compared by research staff, examining what was confirmatory, dissonant, or novel, until agreement was reached about the interpretation of both sets of data together (Askun & Cizel, 2020). The mixed analysis results informed the Discussion.

### Ethical Approval

The project was approved by the Aboriginal Health and Medical Research Council (AH&MRC) and the University of New South Wales, Australia (HREC).

We have not registered a study protocol; however, it was published (Reilly et al., 2023). We aim to submit our data to the Australian Data Archive so it can be accessed with our permission.

## Results

### Overall Change in GEM and K6 Scores

Table 1 presents the pre-, post-, and change scores for the GEM and K6 total scores. Both groups demonstrated some improvement in GEM scores; however, the intervention group showed a larger increase (mean change = 0.67, 95% CI [ –0.05, 1.38]) compared to the control group (mean change = 0.29, [–0.05, 0.62]). For K6, the control group showed a modest increase in distress (mean change = 0.37, [–0.24, 0.99]), while the intervention group showed a slight reduction (mean change = –0.11, [–0.64, 0.41]). However, neither difference was statistically significant (GEM: *p* = .36; K6: *p* = .26).

**Table 1.** Pre-, Post-, and Change Scores on the GEM and Kessler 6 Scales.

Group	GEM pre [95% CI]	GEM post [95% CI]	GEM change [95% CI]	K6 pre [95% CI]	K6 post [95% CI]	K6 change [95% CI]	Significance [between-group]
Control	3.06 [2.98, 3.14]	3.35 [3.04, 3.66]	0.29 [–0.05, 0.62]	1.56 [1.29, 1.84]	1.94 [1.33, 2.55]	0.37 [–0.24, 0.99]	
Intervention	3.33 [3.01, 3.66]	4.00 [3.41, 4.59]	0.67 [–0.05, 1.38]	1.59 [1.18, 2.00]	1.48 [1.17, 1.79]	–0.11 [–0.64, 0.41]	GEM: *p* = .36; K6: *p* = .26

*Note.* Values are means with 95% confidence intervals.

*p*-values refer to independent samples *t*-tests comparing change scores between intervention and control groups.

GEM = Growth and Empowerment Measure.

### Item-Level Outcomes

Table 2 displays pre-, post-, and change scores at the item level for GEM and K6. Among GEM items, the largest positive changes in the intervention group were observed for “Centred & Focused” (mean change = 0.89, 95% CI [0.37, 1.41]), “Happy” (mean change = 0.78, [0.26,1.30]), and “Confidence” (mean change = 0.73, [0.21, 1.25]). However, these differences were not statistically significant when compared with the control group (all *p* > .05).

**Table 2.** Item-Level Pre-, Post-, and Change Scores with Between-Group Comparisons.

Item	Pre-mean (control)	Post-mean (control)	Change (control, 95% CI)	Pre-mean (intervention)	Post-mean (intervention)	Change (intervention, 95% CI)	Between-group *p*-value
Knowledge	3.12	3.5	0.38 [−0.17, 0.93]	3.33	3.6	0.27 [−0.25, 0.79]	.602
Energetic	3.0	3.38	0.38 [−0.17, 0.93]	3.22	4.0	0.78 [0.26, 1.30]	.528
Happy	3.12	3.38	0.26 [−0.29, 0.81]	3.22	4.0	0.78 [0.26, 1.30]	.293
Admired and Valued	3.0	3.38	0.38 [−0.17, 0.93]	3.11	3.9	0.79 [0.27, 1.31]	.528
Speak Out	3.0	3.38	0.38 [−0.17, 0.93]	3.33	4.0	0.67 [0.15, 1.19]	.730
Belonging	3.25	3.38	0.13 [−0.42, 0.68]	3.33	4.0	0.67 [0.15, 1.19]	.308
Confidence	3.0	3.25	0.25 [−0.30, 0.80]	3.67	4.4	0.73 [0.21, 1.25]	.328
Centred and Focused	3.0	3.25	0.25 [−0.30, 0.80]	3.11	4.0	0.89 [0.37, 1.41]	.249
Safe and Secure	3.0	3.25	0.25 [−0.30, 0.80]	3.56	4.1	0.54 [0.02, 1.06]	0.645
Anger	3.12	3.38	0.26 [−0.29, 0.81]	3.44	4.0	0.56 [0.04, 1.08]	.574
Nervous	1.5	1.88	0.38 [−0.17, 0.93]	1.67	1.6	−0.07 [−0.59, 0.45]	.432
Restless/Jumpy	1.62	1.88	0.26 [−0.29, 0.81]	1.56	1.5	−0.06 [−0.58, 0.46]	.478
Struggle	1.75	2.0	0.25 [−0.30, 0.80]	1.56	1.7	0.14 [−0.38, 0.66]	.948
Sad	1.38	1.88	0.50 [−0.05, 1.05]	1.67	1.8	0.13 [−0.39, 0.65]	.579
Worthless	1.5	1.88	0.38 [−0.17, 0.93]	1.56	1.7	0.14 [−0.38, 0.66]	.743
Angry	1.62	2.12	0.50 [−0.05, 1.05]	1.56	1.9	0.34 [−0.18, 0.86]	.765

For the K6 scale, both groups showed small or negligible changes on individual items. None of the between-group comparisons reached statistical significance, and change score confidence intervals generally included zero, indicating a lack of robust intervention effect across individual K6 indicators.

### Summary of Between-Group Comparisons

Although trends in the intervention group suggest improvements in several SEWB indicators, including feelings of energy, confidence, belonging, and reduced anger, these improvements were not statistically greater than those observed in the control group. This is likely due to the modest sample size and variability in responses. Future larger-scale trials are warranted to confirm these effects.

## Qualitative Findings

Three dominant themes from the intervention group, compared with the control group, emerged from our qualitative data analysis. 1. New sense of pride and self-esteem 2. Desire to help other dads. 3. Parenting after ED strengthens the couple relationship.

### Theme 1: Stronger Sense of Pride and Self-Esteem Than Those in the Control Group

Dominant qualitative findings indicated an ED group level “increased pride and self-esteem.”

One participant talked about his and other dads’ feelings after the program, particularly how much more confidence they had, and how much their self-esteem improved. He said: “It (ED) makes you confident and improves our self-esteem.” Another man without prompting said after the program that it improved his self-worth because it increased his awareness of how important his role as a dad was, and that his confidence in parenting had the great benefit of feeling closer to his child: “It makes you feel strong and empowered, and feeling loved by your children, is the best feeling.”… The men repeatedly expressed how the ED program helps them to feel like proud dads because it refocused them on the role, they can play in improving their children’s lives. “I am proud of my children, for getting scholarship to attend boarding school. This makes me feel very proud as a dad … teaching your kids to dream big.”…

### Theme 2: Desire to Help Other Dads

This qualitative theme of a “desire to help other dads” was evident from the qualitative interviews. Men talked about how they could help other young men and new dads to value being proud and capable dads. One man said:

“Young fathers, who don’t know about parenting, this program could help them, and with the support of local experienced dad’s, creating a space place for this program for them, and also, talk to them (young first-time dad’s) before they’re due to have the baby, would be a good thing.”…

Men valued the program in their community such that they started to plan how to ensure it was continued as a regular program: “If this program is delivered here, it will be successful. I can see it run through the men’s group; they need good programs like this too.”

### Theme 3: Parenting Strengthens the Couple Relationship

We found that the relationship with the men’s intimate partner improved because of the ED. The improvement was described as having a better understanding of the partner’s experiences of parenting, seeing how better knowledge of parenting was valued by his partner, and feeling the positive emotional impacts of playing a role in seeing his partner happier.

“I …understand more about what it takes to manage my children, and this makes my partner happy”…“When I come home from work, some days I will take our kids for a drive on country, for a few hours to give my partner a break”…“Being a engaged father, takes the stress off her”…“by being both involved in the caring of our children, you both have a stake in your children’s upbringing”…“Happy wife – Happy life, they say, and it is true”…

## Discussion and Conclusions

ED included a novel feasibility RCT with a qualitative component, implemented by local First Nations community-based researchers. The RCT undertaking, with training and support provided by external Indigenous and non-Indigenous researchers, proved successful, with strict application of all procedures ensuring rigour and reliability of findings. There was also a high level of First Nations researcher and community approval and support for the RCT program and its application to generate useful insights and results. On the strength of the qualitative findings, the mixed methods results suggest that the ED intervention had a positive impact on men’s SEWB, contributing to evidence that ED may address an urgent need to reduce the serious equity gap in First Nations men’s mental health. A subsequent large-scale evaluation of ED is needed to confirm the effectiveness of ED quantitatively. The use of the RCT and qualitative methods strengthened capacity in communities to conduct research that may contribute evidence to sustain effective programs over time.

The quantitative results from the feasibility RCT were unable to empirically confirm the effectiveness of the program, a result which is likely due to a lack of power. This indicates that larger trials are needed, and we now have locally trained researchers in Aboriginal communities able to conduct research of that nature in the future. Although not statistically significant, the ED intervention, that included a focus on learning and sharing experiences of the father role, and practicing engaged parenting with their adolescent, indicated a trend toward positive change on items including feeling energetic, having a sense of belonging in the community, greater confidence, feeling centred and focused, and reduced anger compared to the control group. These items are consistent with clinical indicators for improved metal health, or SEWB. The findings are provided with even greater meaning when considered in the context of the qualitative interview data, where men said that they experienced a stronger sense of pride and self-esteem associated with their identification and self-perception of being dads, and a new way of thinking about the importance of their role as dads on improving the well-being and lives of their children. SEWB in men strengthened with the recognition of how valuable they are to their family and community.

The qualitative data support the quantitative insights that those in the ED intervention experienced greater personal confidence, which gave them more energy and helped them to feel more centred and focused compared with those in the control group. This suggests that future focus on the value and importance of male parenting in First Nations communities, particularly when undertaken as part of a systematic program such as ED, is a significant factor in strengthening SEWB and helping to bridge the gap in mental health inequality experienced by First Nations men. Whereas employment options and other avenues for providing meaning and confidence amongst men might also have a positive effect on SEWB, most men will be parents, and it is therefore one role that is ubiquitous and consequential, making it a highly feasible area for mental health interventions.

The finding related to the desire to help other dads is theoretically connected to the quantitative trial data, particularly resonating with the ED group finding of participants feeling more admired and valued by others, presumably through their greater sense of value as a knowledgeable parent who can support other dads. The trial data showed men in the ED group also had a greater sense of belonging in the community, and we anticipate that this was because the men experienced a sense of value in themselves, which, in a collectivist society, necessarily leads to the desire to share that experience to support other dads. Men who were engaged with helping other men therefore felt more connected rather than alienated from the wider community. Men in the ED group also felt more outspoken compared to the control group, another possible indicator of feeling more empowered and confident to share their views and perspectives in the context of their newfound confidence in the value of their role as a dad.

We found that the relationship with their intimate partner improved. The men in the ED group, for example, described having a better understanding of their partner's experiences of parenting. The men noted their partner’s appreciation for thier new engagement with the family and parenting role, and felt the positive emotional impacts of seeing thier partner happier. The finding of a strengthened intimate partner relationship is unique in the context of literature related to parenting in First Nations communities. These findings together with the documented reduction in feelings of anger amongst men in the ED indicate that the intervention could be useful in reducing the risk for family conflict and potentially intimate partner violence (IPV) (Andersson & Nahwegahbow, 2010; Bulman & Hayes, 2011; Reilly et al., 2023). IPV is a significant and concerning public health and human rights issue in First Nations communities, and this study indicates the need to further study this intervention and its potential role in reducing risk of relationship stress and couple violence.

Limitations of this feasibility study are that the randomised controlled trial data and the descriptive analysis of items in the measures can only be indicative. The RCT recruitment process was not randomised, although the recruited sample was randomised to the intervention or control group. The sample size is small, limiting the findings to empirically derived but indicative insights. The data are strengthened by the qualitative interviews, which provided depth and insight into the quantitative findings. A profoundly valuable outcome is that community-based researchers have new skills and are able to conduct rigorous design evaluations, and to produce data that can be applied to sustain projects that they feel are useful and beneficial to their communities and potentially, if extended across other First Nations populations, to address the national gap in mental health inequalities.

## Appendix A

### Community demographic and map

#### Doomadgee

Old Doomadgee was settled as a Christian mission in 1933, on the coast of the Lower Gulf of Carpentaria at Point Parker, known to the Traditional Owners as Dumji. In 1936 the Old Doomadgee mission was flattened by a tropical cyclone, and a more suitable site was established inland, at the current location on the banks of the Nicholson River. There are two main peoples (tribes) coexisting on this land: Waanyi and Ganggalidda. The Doomadgee mission ceased operation in the early 1980s and was handed over to the Queensland Government, becoming a Deed of Grant in Trust (DOGIT) community. Since 2005, the Doomadgee community has become a Shire Council, with local people (Waanyi and Ganggalidda) becoming councillors leading policies and practices for a current population of 1405. Doomadgee is located 992 km west of Cairns in Far North Queensland.

#### Wujal Wujal

Wujal Wujal, so nice you have to say it twice, (the place of many waterfalls). Wujal Wujal is a discrete First Nations community, govern by the Wujal Wujal Aboriginal Shire Council, located in the Bloomfield River upper Daintree, Cape York region Queensland. In 1881, the Bloomfield River Lutheran mission was established on land belonging to the KuKu-Yalanji people. In 1964, land approximately 6 kilometres upstream from where the old mission had been, was declared an Aboriginal reserve. The land was cleared for growing crops, a manager’s house, a boy’s dormitory, a mess hut and saw bench were constructed on this site. Subsequently, all the camps were consolidated into this one, currently Wujal Wujal community. Wujal Wujal is part of the traditional homelands of the Eastern Kuku (Goo-goo) Yalanji (Ya-lan-gee) people. In the 2021 census, the locality of Wujal Wujal had a population of 276 people. Additionally, Wujal Wujal is located approximately 30 kilometres north of Cape Tribulation, 60 kilometres south of Cooktown, and 347 kilometres north of Cairns.

#### Hope Vale

Hope Vale is a town within the Aboriginal Shire of Hope Vale and is the oldest continuing mission community in North Queensland. In addition, Hope Vale, is a missionary of the Lutheran Church, established the Elim Aboriginal Mission on the beach of the north shore of Cape Bedford and the Cape Bedford Mission (1886) nearby. Nonetheless, while originally thriving, the Elim's mission future became unsustainable, due to several reason including weather, and the people were relocated to Hope Vale. Hope Vale traditional language group are the Guugu Yimithirr people, land encompassing Hope Vale, Cooktown, Cape Bedford, Battle Camp, and sections of the Normanby River and Annan River. The population is 1,004, and located 46 kilometres Northwest of Cooktown, and 372 kilometres North of Cairns Far North Queensland.
